# Supplementary material for: Single cell deciphering of progression trajectories of the tumor ecosystem in head and neck cancer
Source: Nat Commun. 2024 Mar 22;15:2595. doi: 10.1038/s41467-024-46912-6 (PMC10959966; doi:10.1038/s41467-024-46912-6)
Supplement: Supplementary file 1 — Supplementary Information [file 41467_2024_46912_MOESM1_ESM.pdf]

Supplementary Information for:

Single-cell Deciphering of Progression Trajectories of the Tumor Ecosystem in Head and Neck Cancer

Authors:

Liu ZL[1#], Meng XY[1#], Bao RJ[2#], Shen MY[2], Sun JJ[3], Chen WD[4], Liu F[5], He Y[1]*

Affiliations:

1. Department of Oral Maxillofacial & Head and Neck Oncology, Shanghai Ninth People's Hospital, Shanghai Jiao Tong University School of Medicine; College of Stomatology, Shanghai Jiao Tong University; National Center for Stomatology; National Clinical Research Center for Oral Diseases; Shanghai Key Laboratory of Stomatology Shanghai 200011, P.R. China.
2. Shanghai Institute of Immunology, State Key Laboratory of Oncogenes and Related Genes, Department of Immunology and Microbiology, Shanghai Jiao Tong University School of Medicine, Shanghai 200025, China.
3. Department of Oral Pathology, Shanghai Ninth People's Hospital, Shanghai Jiao Tong University School of Medicine; College of Stomatology, Shanghai Jiao Tong University; National Center for Stomatology; National Clinical Research Center for Oral Diseases; Shanghai Key Laboratory of Stomatology Shanghai 200011, P.R. China.
4. Novel Bioinformatics Co., Ltd, Shanghai, P. R. China.
5. Department of Oncology, Shanghai Ninth People's Hospital, Shanghai Jiao Tong University School of Medicine; Shanghai 200011, P.R. China

# Liu ZL, Meng XY, and Bao RJ have contributed equally to this work.

Correspondence to Prof. Yue He DDS, MD, PhD;

Department of Oral Maxillofacial & Head and Neck Oncology, Shanghai Ninth People's Hospital Affiliated to Shanghai Jiao Tong University School of Medicine, Shanghai, National Center of Stomatology, National Clinical Research Center for Oral Disease, China, 200011.

Tel: +86(0)21 23271699×5656

Email: william5218@126.com

This PDF file includes:

Supplementary Figure 1-6

Supplementary References

Supplementary Fig1

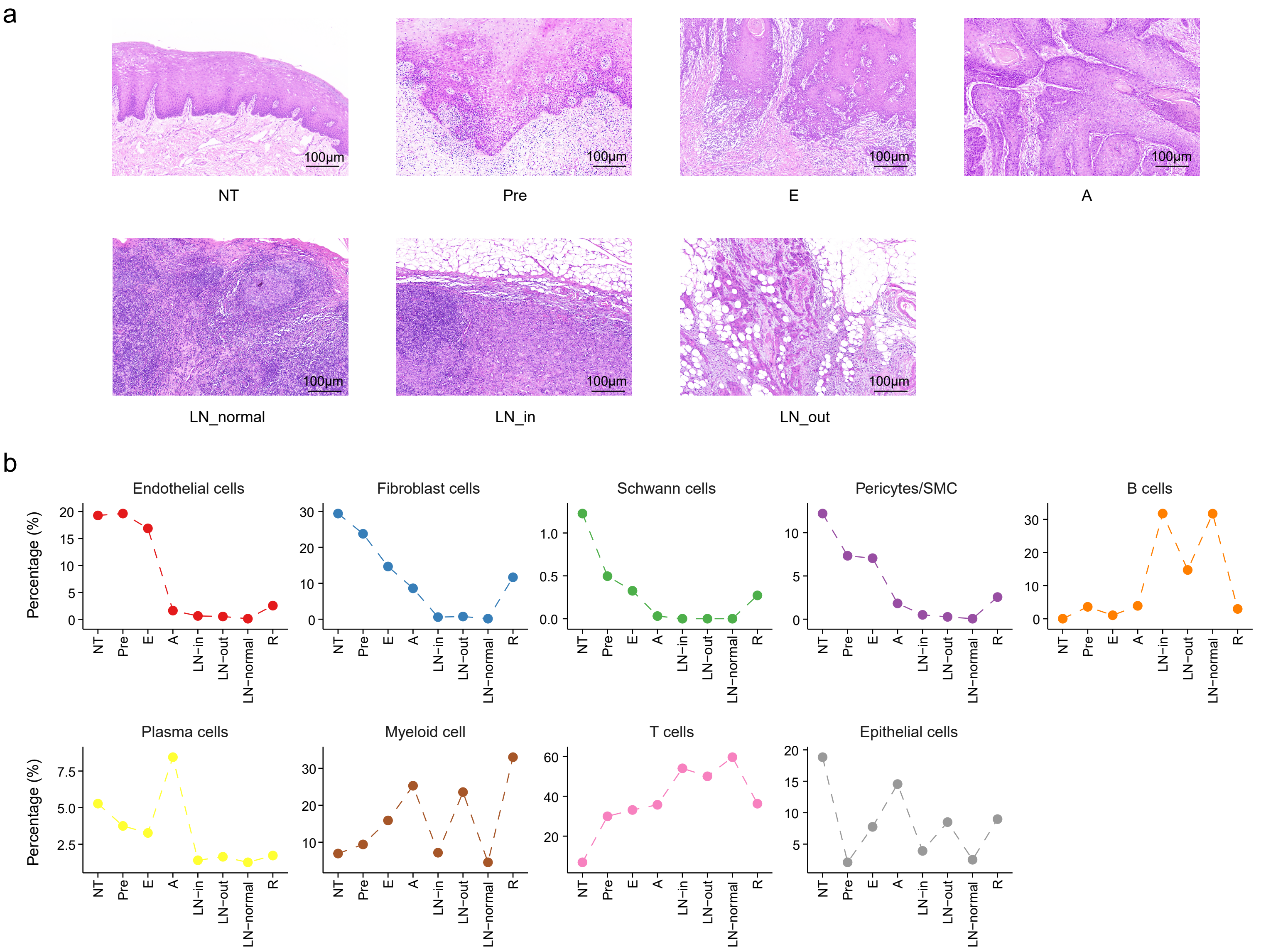

Supplementary Fig1. Overview of HNSCC samples, related to Figure 1. (a) Representative H&E staining of tissue samples biopsied for scRNA-seq in different stages during HNSCC initiation and progression. Scale bar = 100μm. (b) Line charts show the dynamic proportion change of main cell clusters in different tumor stages. Cell numbers are shown below. Endothelial: NT: 3377, Pre: 3281, E: 2435, A: 472, LN-in: 69, LN-out: 62, LN-normal: 10, R: 299; Fibroblast: NT: 5150, Pre: 3974, E: 2117, A: 2510, LN-in: 65, LN-out: 89, LN-normal: 13, R: 1373; Schwann: NT: 215, Pre: 83, E: 47, A: 9, LN-in: 0, LN-out: 0, LN-normal: 0, R: 32; Pericyte/SMC: NT: 2140, Pre: 1226, E: 1018, A: 533, LN-in: 55, LN-out: 32, LN-normal: 5, R: 301; B: NT: 3, Pre: 603, E: 153, A: 1140, LN-in: 3469, LN-out: 1742, LN-normal: 2718, R: 349; Plasma: NT: 924, Pre: 627, E: 472, A: 2465, LN-in: 153, LN-out: 194, LN-normal: 108, R: 203; Myeloid: NT: 1215, Pre: 1572, E: 2294, A: 7381, LN-in: 782, LN-out: 2786, LN-normal: 390, R: 3886; T: NT: 1202, Pre: 5009, E: 4783, A: 10428, LN-in: 5899, LN-out: 5914, LN-normal: 5105, R: 4269; Epithelial: NT: 3301, Pre: 351, E: 1118, A: 4248, LN-in: 428, LN-out: 1005, LN-normal: 215, R: 1056. Source data are provided as a Source Data Fig. S1b.

Supplementary Fig2

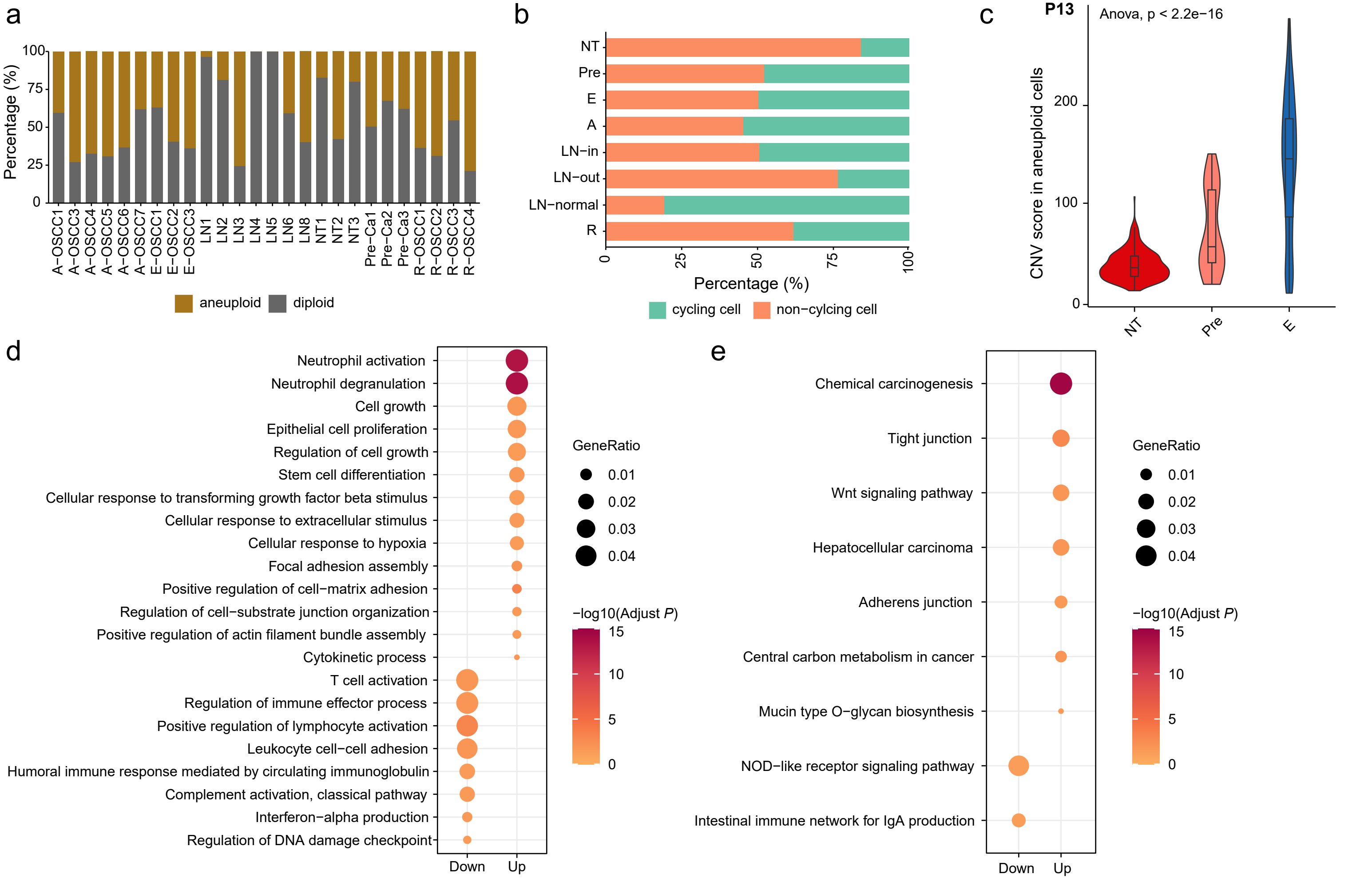

(To be continued)

Supplementary Fig2

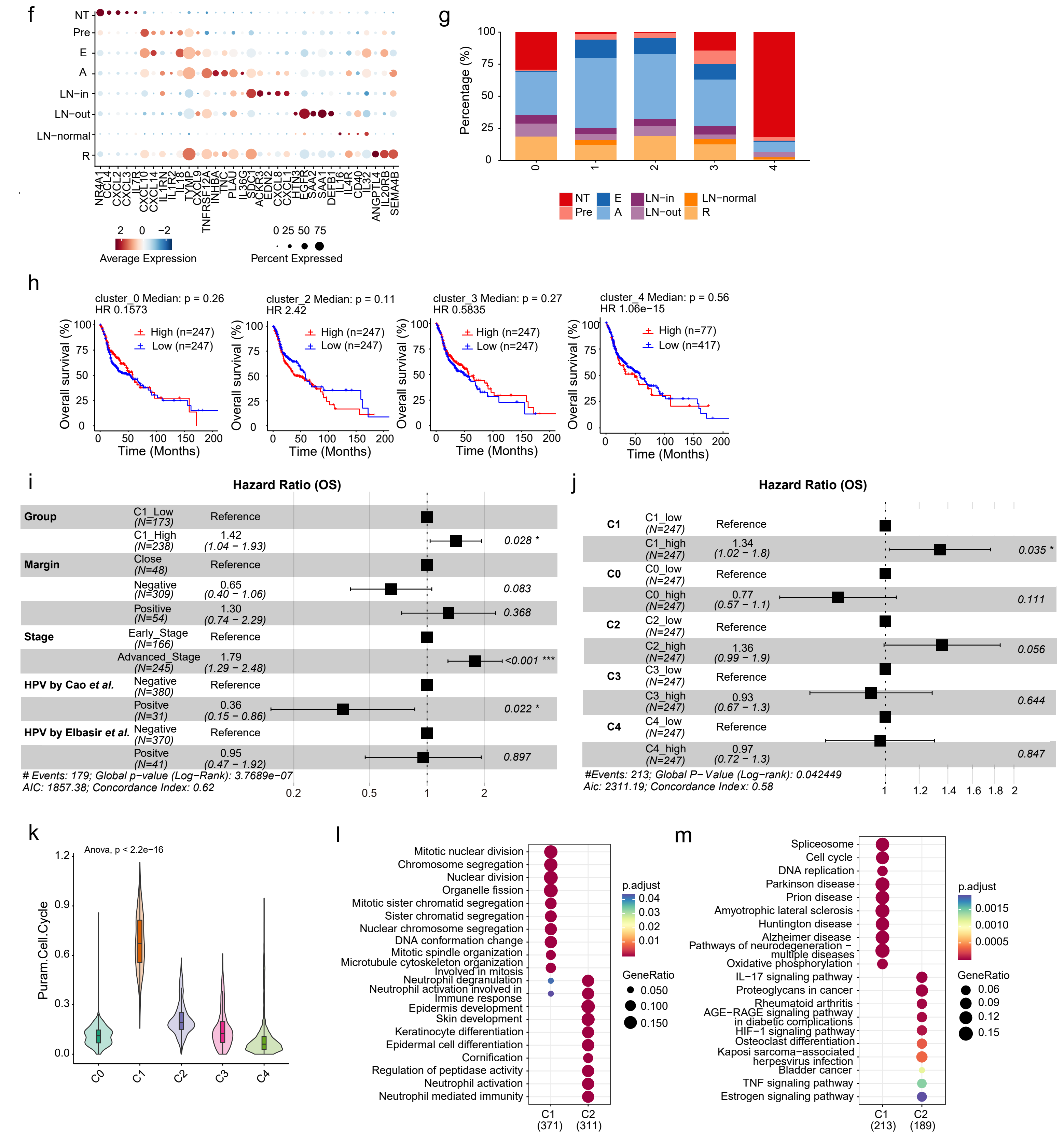

(To be continued)

Supplementary Fig2

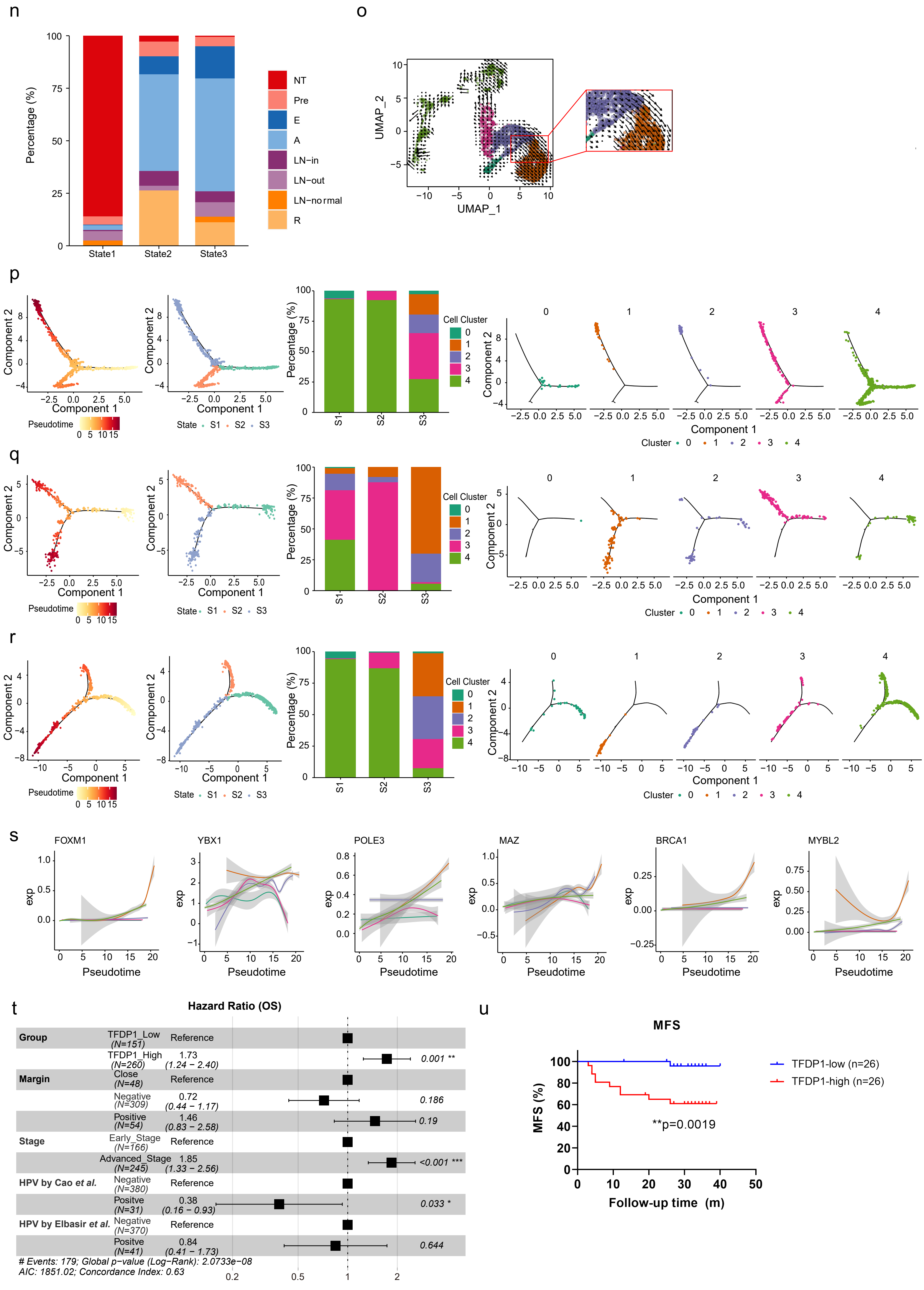

Supplementary Fig2. Epithelial cells during HNSCC progression, related to Figure 2. (a-b) Bar chart shows percentage of aneuploid and diploid epithelial cells in different samples (a) and cycling and non-cycling cells in each group (b). (c) Violin plots show CNV scores of aneuploid epithelial cells of P13. (d-e) Bubble plots of GO (d) and KEGG (e) pathways for differentially expressed genes (DEGs) in aneuploid compared to diploid epithelial cells from NT samples of P13. (f) Dot plot shows average expression of cytokines in epithelial cells. (g) Bar chart shows percentage of cells from different groups in each cluster. (h) The Kaplan-Meier curves of samples with signatures of different clusters in TCGA-HNSCC cohort (n = 494), 247 samples in high- and 247 samples in low-groups for cluster 0/2/3 and 77 samples in the high- and 417 samples in the low-group for cluster 4. (i-j) Multivariate Cox regression model analysis (i) and hazard ratios for overall survival (OS) analysis (j) in TCGA-HNSCC cohort. (k) Violin plots show the scores of cell cycle signature from Puram *et.al*. [1] (l-m) Dot plots of GO (l) and KEGG (m) analysis of DEGs between C1 and C2. (n) Bar chart shows percentage of cells in each cell state. (o) RNA velocity analysis of 7054 epithelial cells from 26 samples. (p-r) Potential trajectory of epithelial cells from P2 (p, 1407 cells), P10 (q, 395 cells), and P13 (r, 1499 cells) inferred by Monocle2. (s) The dynamic changes of TFs. (t) Multivariate Cox regression model analysis in TCGA-HNSCC cohort. (u) The Kaplan-Meier curves of samples with high (n = 26) and low (n = 26) *TFDP1* expression level in validation cohort (n = 52). HPV status signatures in i, j, and t were from Cao *et al.* [2] and Elbasir *et al.* [3] Data are presented as Mean ± SD. *P* values were calculated by Fisher's test and were adjusted by FDR in d, e, l, and m, by one-way ANOVA test in c and k, and by two-sided log-rank test in h, i, j, t, and u. Source data are provided as a Source Data Fig. S2a-n, p-u.

Supplementary Fig3

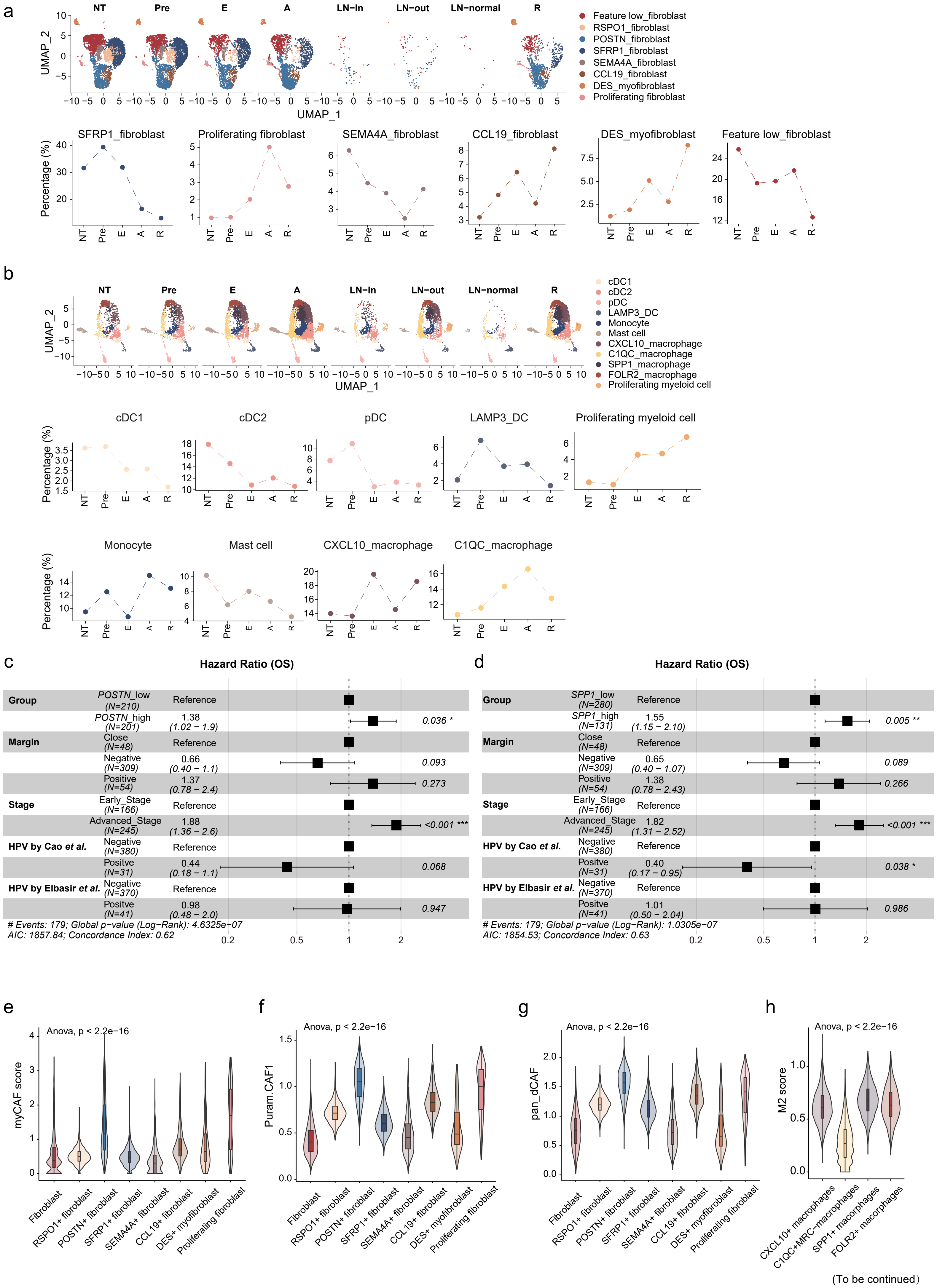

(To be continued)

Supplementary Fig3

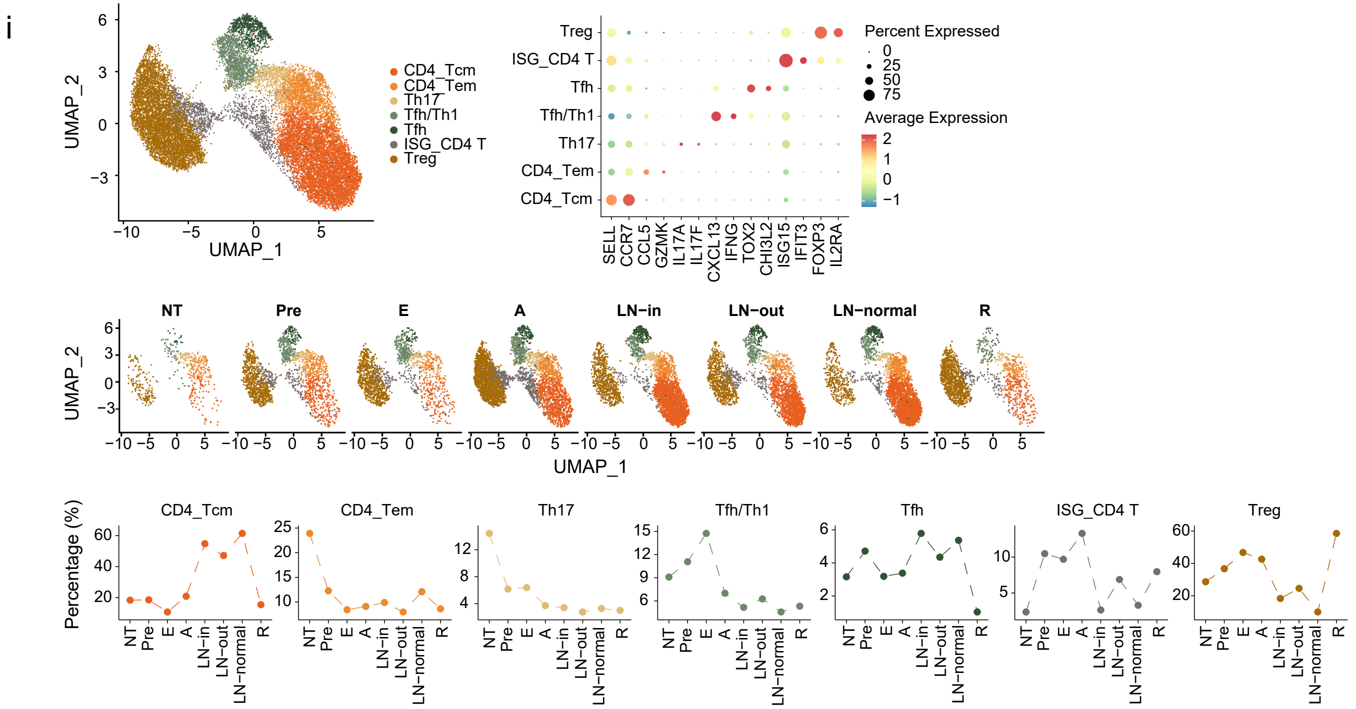

(To be continued)

Supplementary Fig3

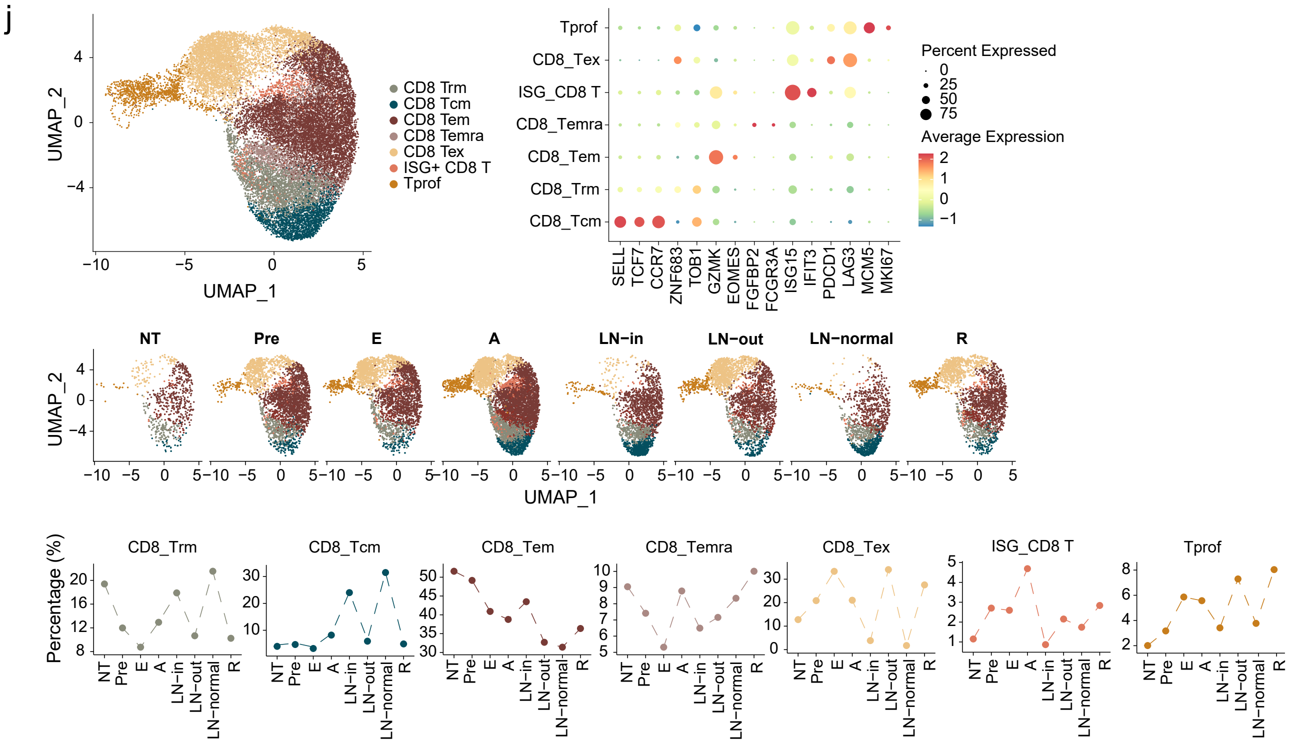

(To be continued)

Supplementary Fig3

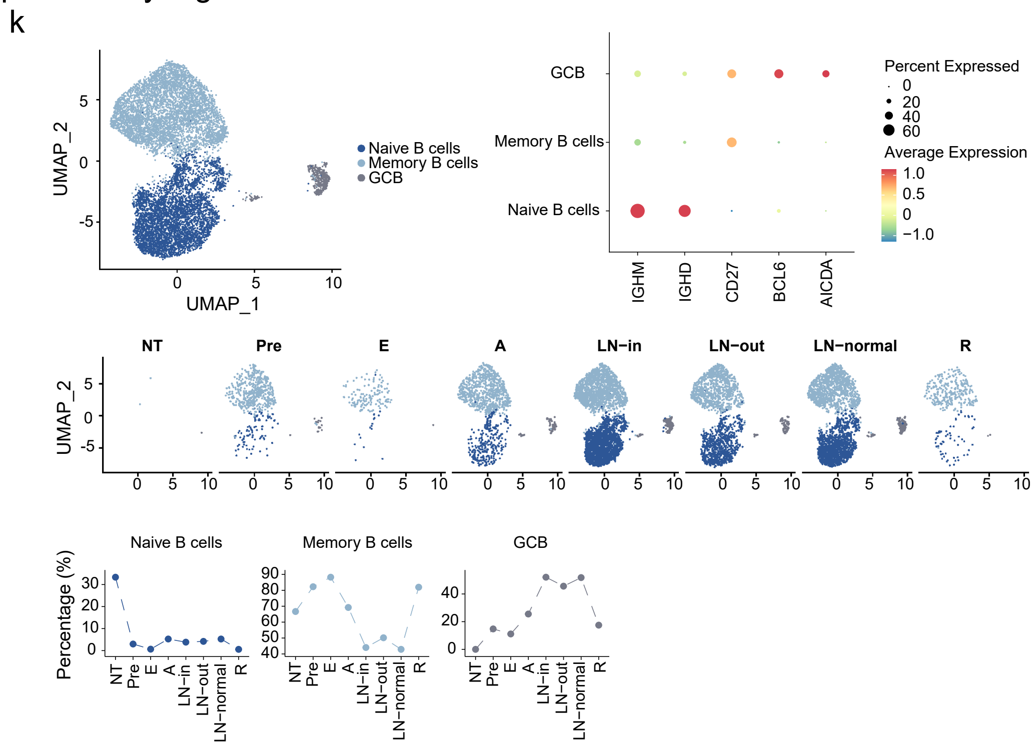

(To be continued）

Supplementary Fig3

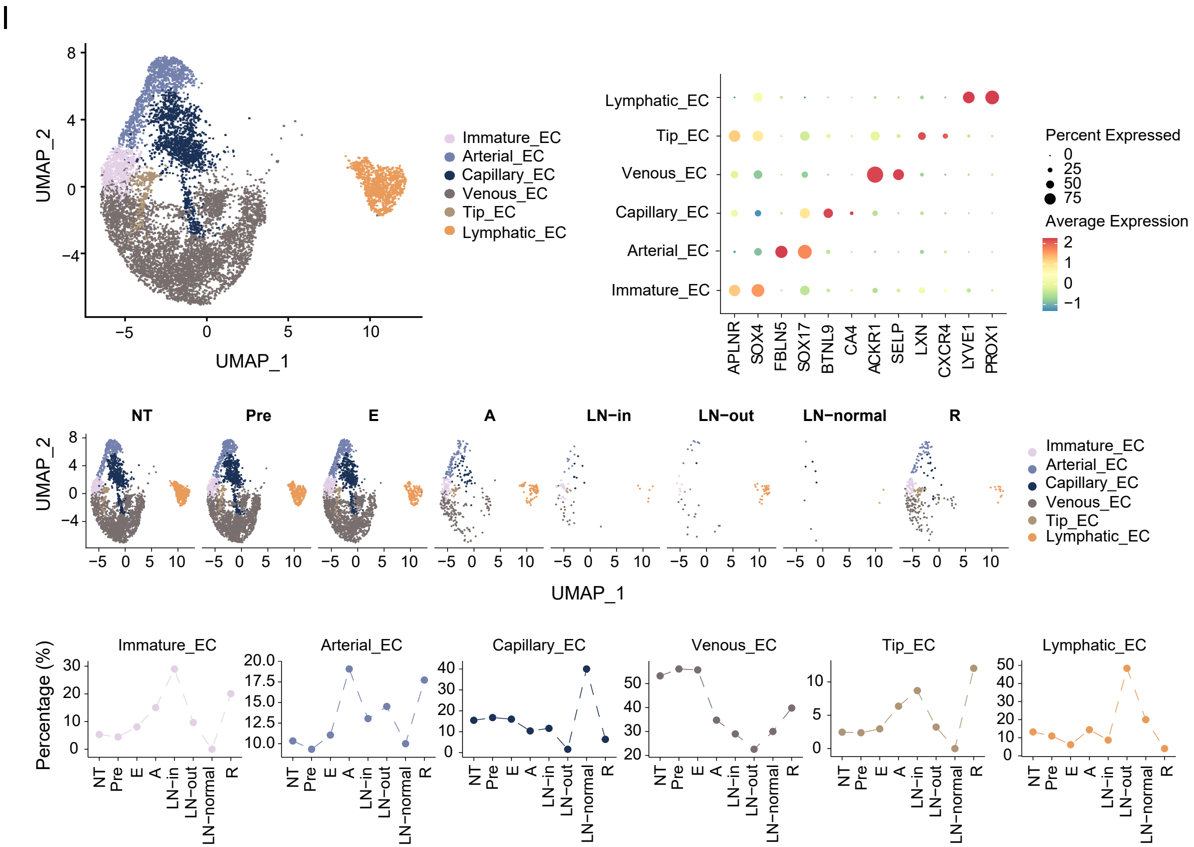

Supplementary Fig3. Characterization of other cell components during HNSCC progression, related to Figure 3. (a) UMAP plots and infiltration proportion of subclusters of fibroblasts (NT: 5150, Pre: 3974, E: 2117, A: 2510, LN-in: 65, LN-out: 89, LN-normal: 13, R: 1373). (b) UMAP plots and infiltration proportion of subcluster of myeloid cells (NT: 1215, Pre: 1572, E: 2294, A: 7381, LN-in: 782, LN-out: 2786, LN-normal: 390, R: 3886). (c-d) Multivariate Cox regression model analysis including the top 50 signature score of *POSTN*$^{+}$ fibroblasts (c) and *SPP1*$^{+}$ macrophages (d) in TCGA-HNSCC cohort. (e-g) Violin plots show the scores of myCAF signature from Elyada *et.al* [4] (e), Puram_CAF1 signature from Puram *et.al* [1] (f), and pan_dCAF signature from Galbo *et.al* [5] (g) of our fibroblast subpopulations. (h) Violin plots show the scores of M2 signature from Cheng *et.al* [6] of our macrophage subpopulations. (i) UMAP plots, marker genes dot plot, and infiltration proportion of subclusters of 21016 CD4^{+} T cells from 26 samples (NT: 506, Pre: 2014, E: 41661, A: 4522, LN-in: 3820, LN-out: 3036, LN-normal: 3726, R: 1731). (j) UMAP plots, marker genes dot plot, and infiltration proportion of subclusters of 21539 CD8^{+} T cells from 26 samples (NT: 696, Pre: 2995, E: 3122, A: 5906, LN-in: 2079, LN-out: 2878, LN-normal: 1379, R: 2538). (k) UMAP plots, marker genes dot plot, and infiltration proportion of subclusters of 10177 B cells from 26 samples (NT: 3, Pre: 603, E: 153, A: 1140, LN-in: 3469, LN-out: 1742, LN-normal: 2718, R: 349). (l) UMAP plots, marker genes dot plot, and infiltration proportion of subclusters of 10005 endothelial cells from 26 samples (NT: 3377, Pre: 3281, E: 2435, A: 472, LN-in: 69, LN-out: 62, LN-normal: 10, R: 299). 3 NT samples, 3 Pre samples, 3 E samples, 6 A samples, 3 LN-in samples, 2 LN-out samples, 2 LN-normal samples, and 4 R samples for related plots. HPV status signatures in c and d were from Cao *et al.* [2] and Elbasir *et al.* [3] Data are presented as Mean ± SD. *P* values were calculated by one-way ANOVA test in e-h and by two-sided log-rank test in c-d. Source data are provided as a Source Data Fig. S3a-l.

Supplementary Fig4

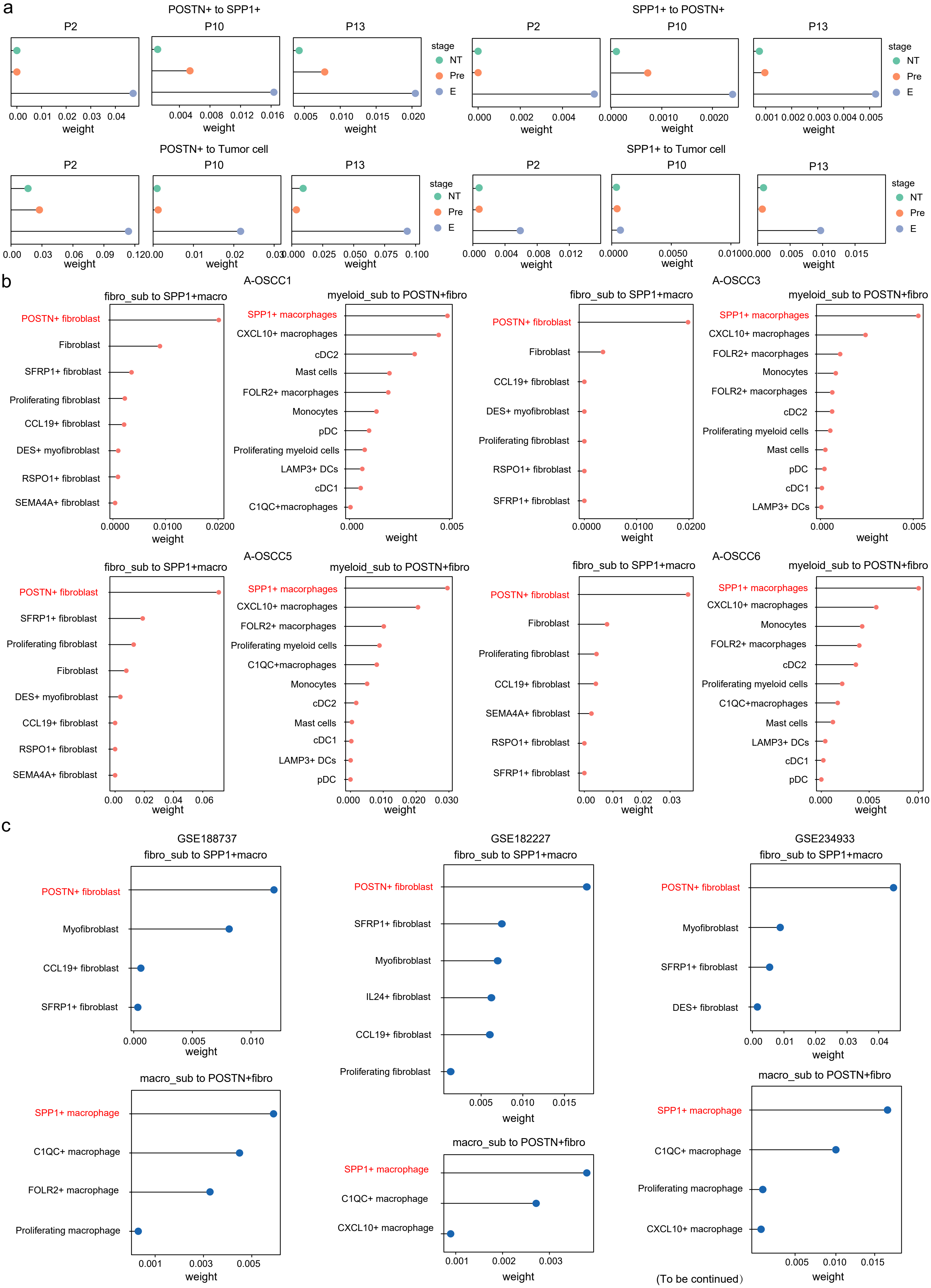

(To be continued)

Supplementary Fig4

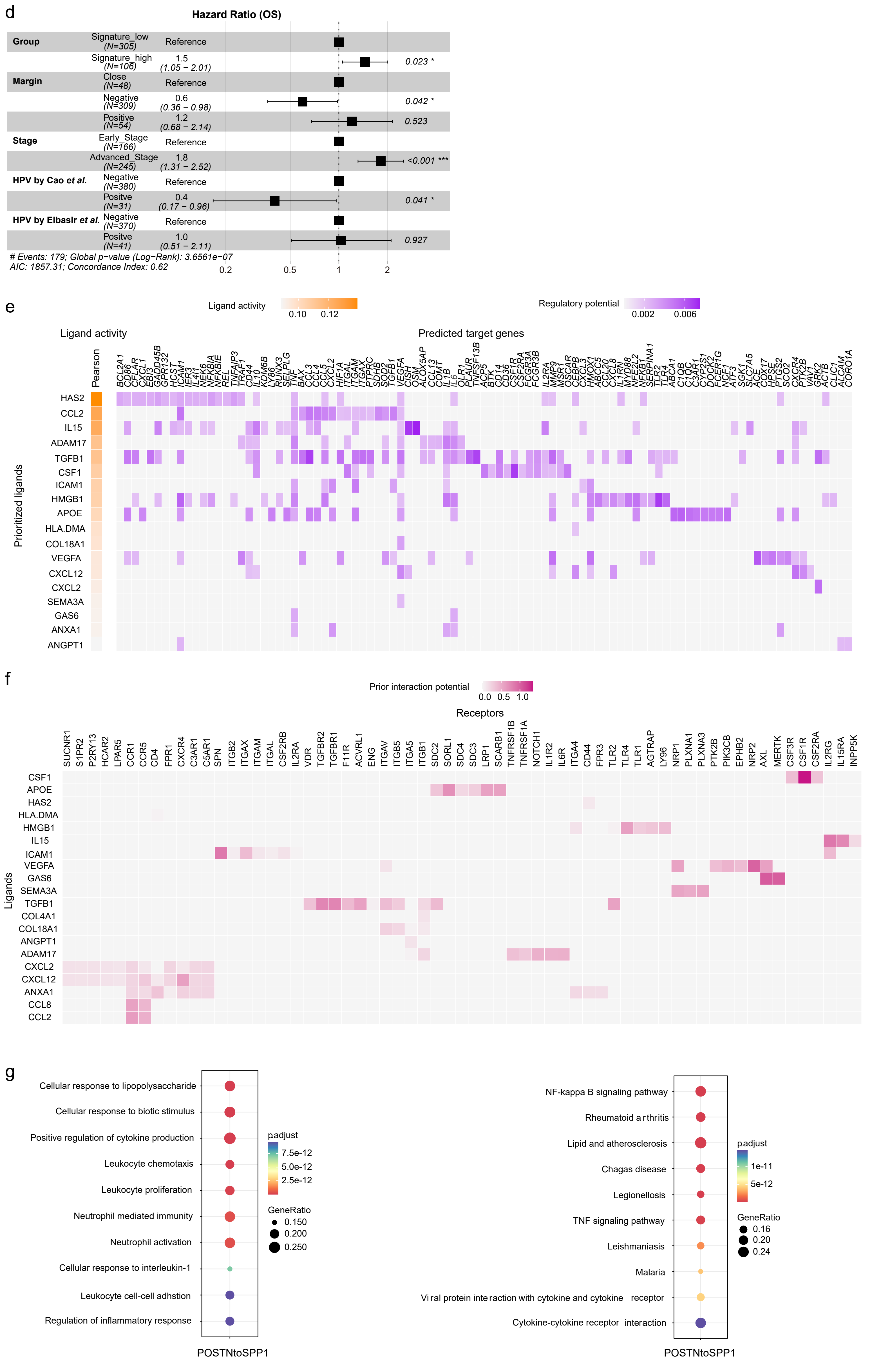

(To be continued)

Supplementary Fig4

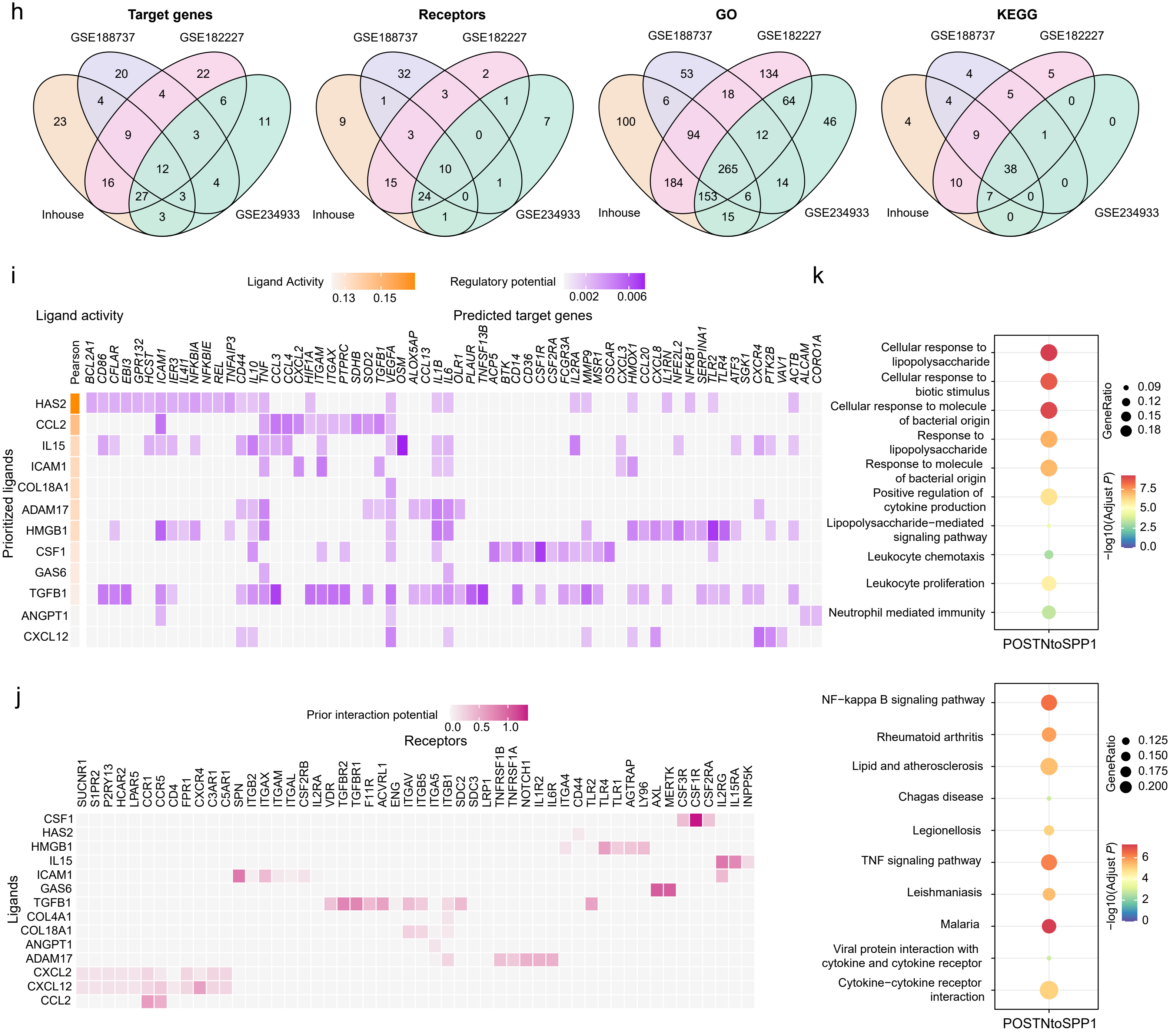

(To be continued)

Supplementary Fig4

l

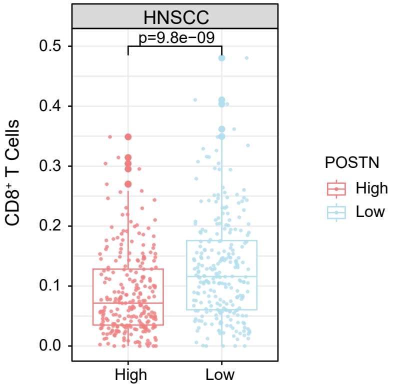

m

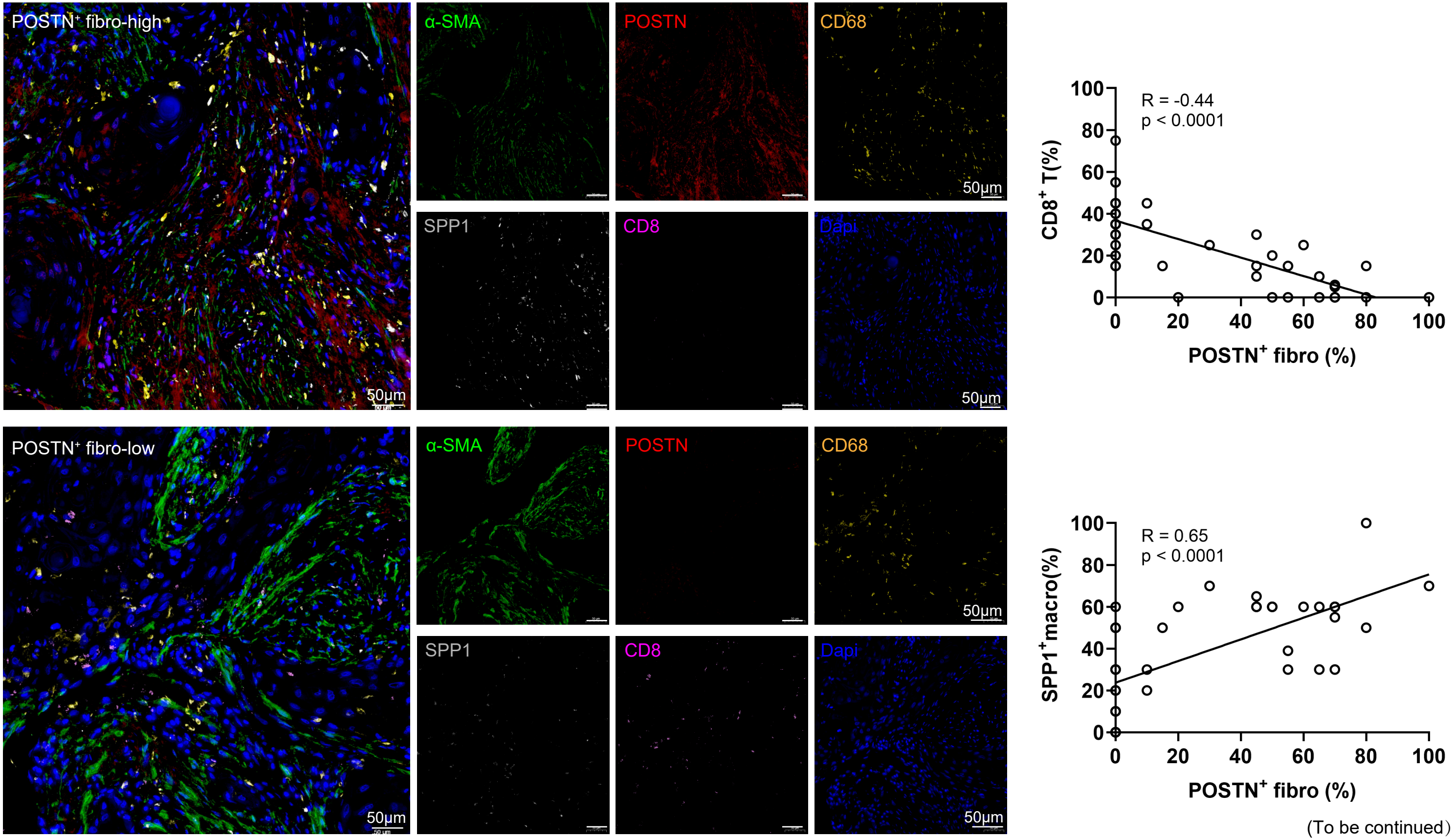

(To be continued)

Supplementary Fig4

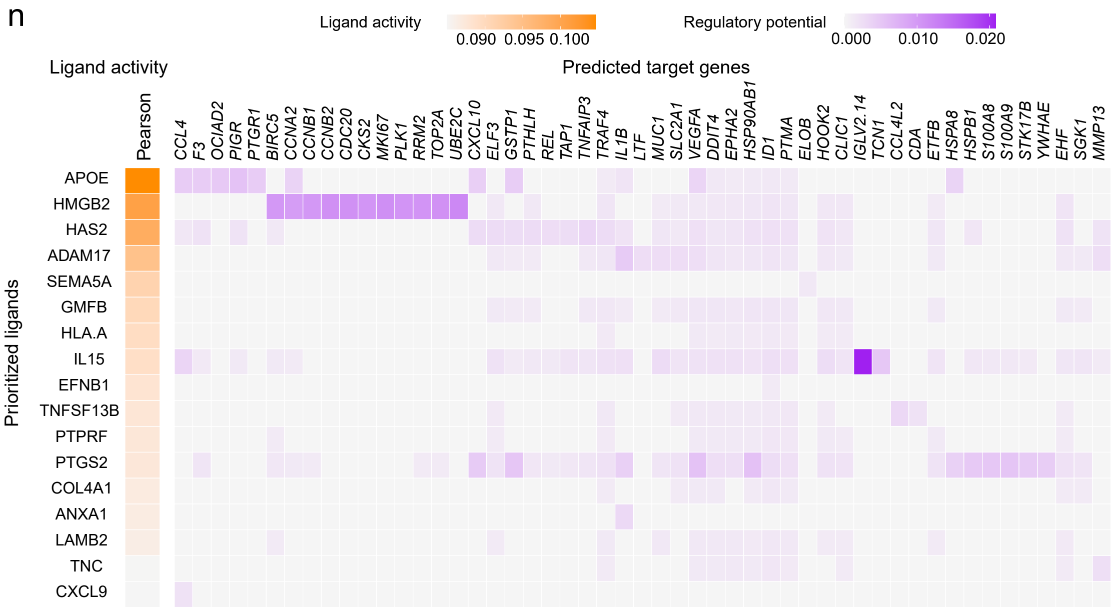

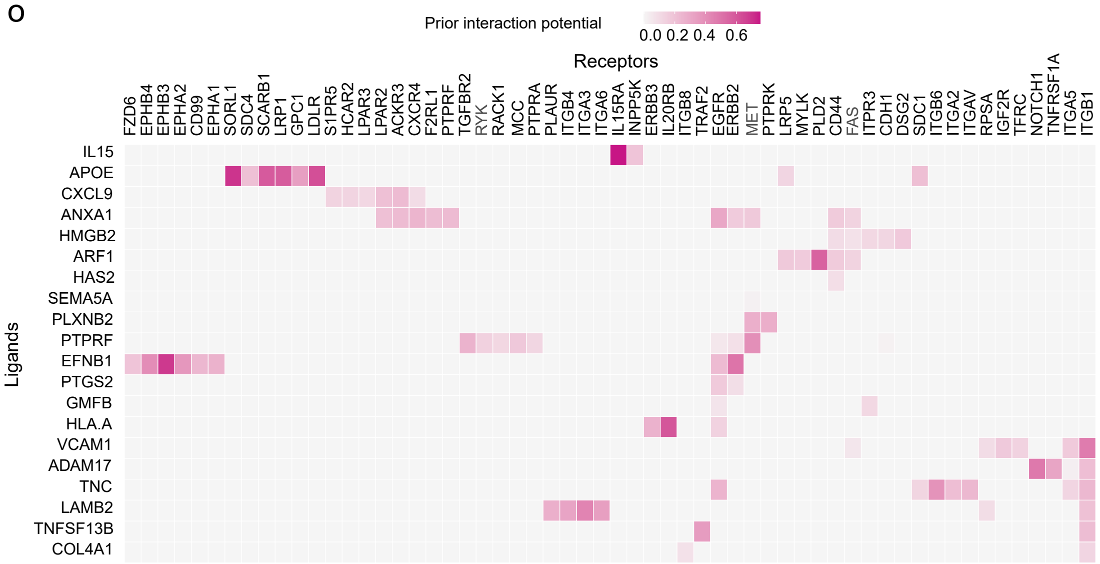

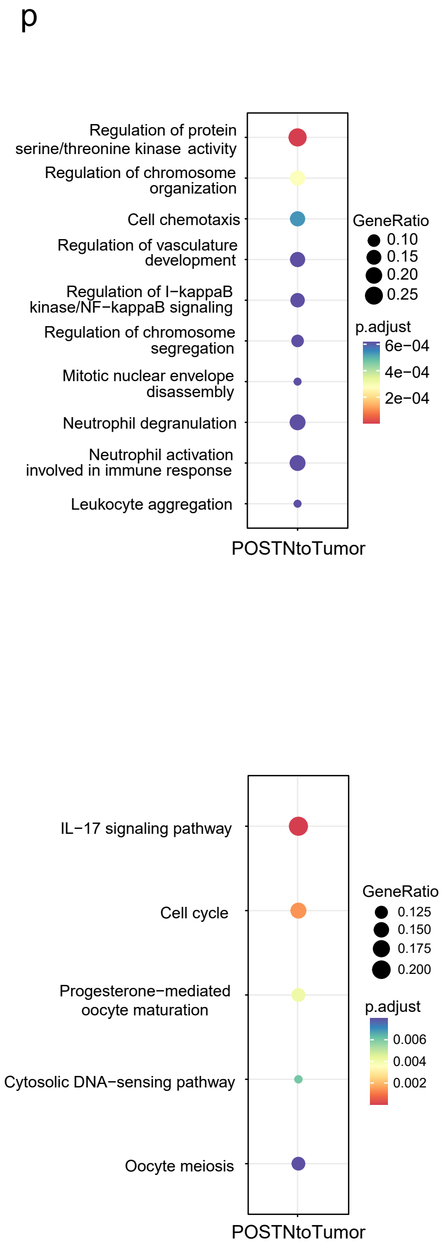

(To be continued)

Supplementary Fig4

q

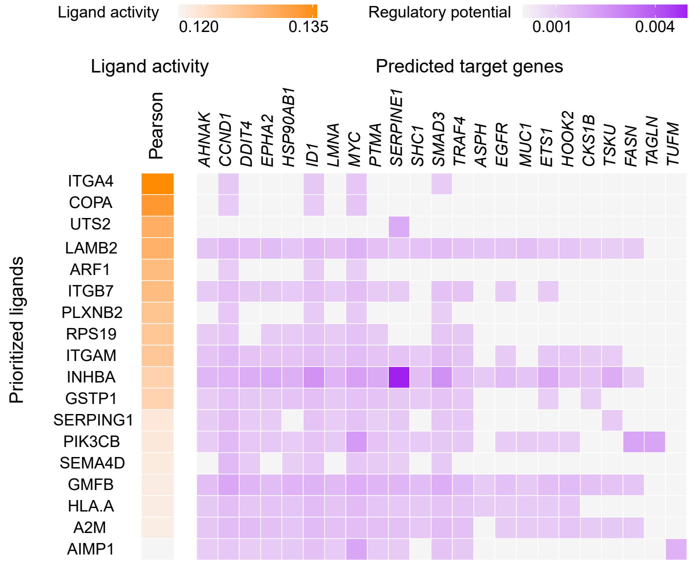

s

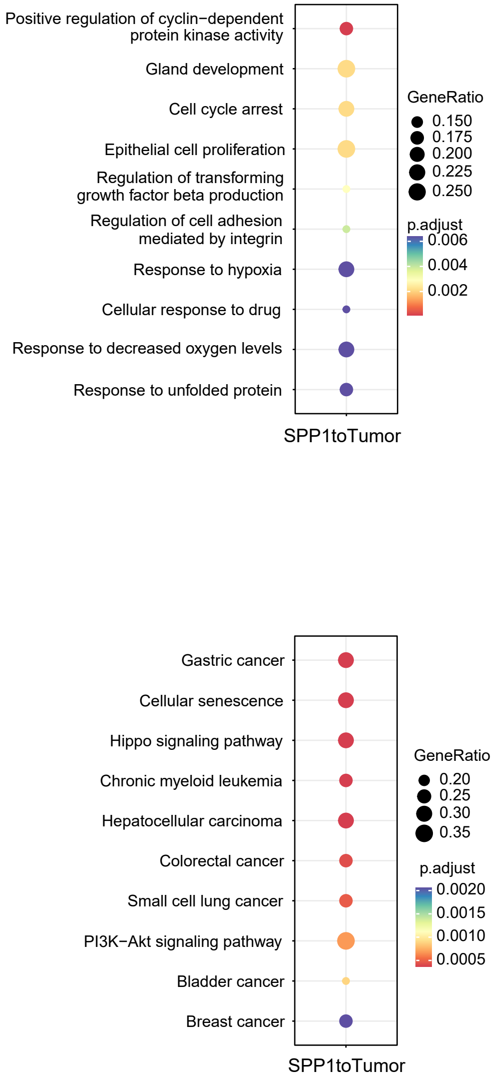

r

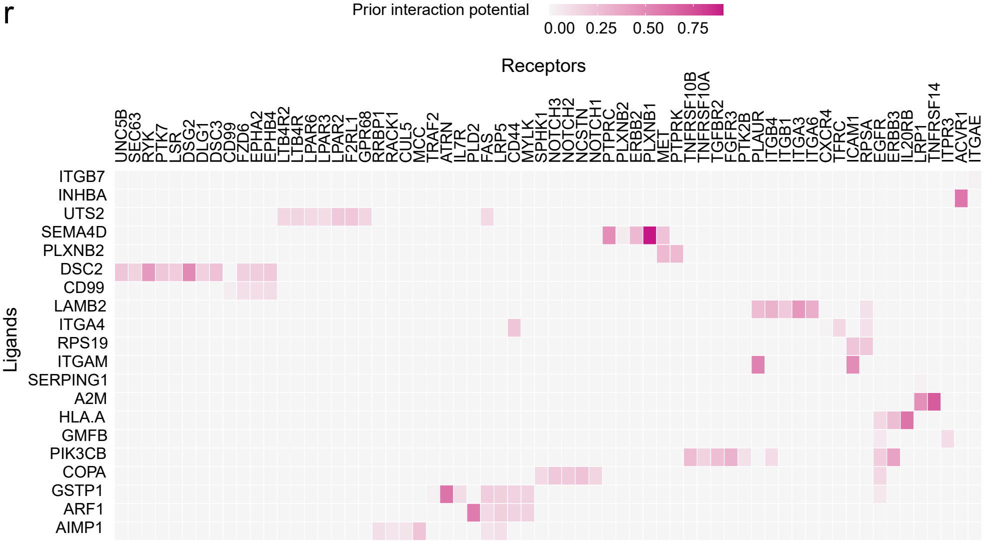

t

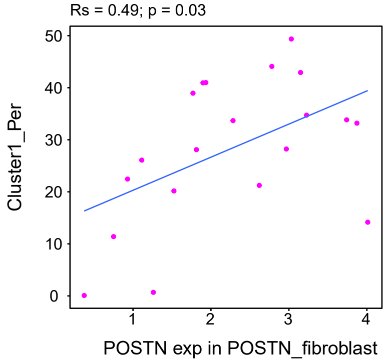

Supplementary Fig4. Interaction among *POSTN*⁺ fibroblasts, *SPP1*⁺ macrophages, and tumor cells during HNSCC progression, related to Figure 4. (a) Lollipop plots show cell-cell interaction strengths of P2/10/13. (b) Lollipop plots show cell-cell interaction strengths of A-OSCC1/3/5/6 samples. (c) Lollipop plots show cell-cell interaction strengths of GSE188737 [7], GSE182227 [8], and GSE234933 [9]. (d) Multivariate Cox regression model analysis in TCGA-HNSCC cohort. HPV status signatures were from Cao *et al.* [2] and Elbasir *et al.* [3] (e-f) Heatmap of Nichenet analysis shows regulatory patterns of *POSTN*$^{+}$ fibroblasts to *SPP1*$^{+}$ macrophages. (g) Representative GO (left) and KEGG pathways (right) enrichment of the predicted target genes expressed in *SPP1*$^{+}$ macrophages. (h) Venn plots show overlapping genes and enrichment pathways among GSE188737 [7], GSE182227 [8], GSE234933 [9], and in-house data inferred by NicheNet algorithm. (i-k) Overlapping ligands (i), target genes (i), receptors (j), target genes GO enrichment pathways (k, upper), and KEGG enrichment pathways (k, lower) between GSE182227 [8] and in-house data. (l) Box plot shows infiltrated CD8^{+} T cells in *POSTN*^{high} (n = 247) and *POSTN*^{low} (n = 248) group in TCGA-HNSCC cohort (n = 495). The boxes show the median ± 1 quartile, with the whiskers extending from the hinge to the smallest or largest value within 1.5× the IQR from the box boundaries. (m) Representative images of mIHC staining of *POSTN*$^{+}$ fibroblasts, *SPP1*$^{+}$ macrophages, and CD8^{+} T cells in HNSCC samples. Scale bar = 50 μm. The Pearson correlation quantitative result is shown on the right. n = 40. (n-o, q-r) Heatmap of Nichenet analysis shows regulatory patterns of *POSTN*$^{+}$ fibroblasts (n-o) and *SPP1*$^{+}$ macrophages (q-r) to tumor cells. (p, s) Representative GO (p and s, upper) and KEGG pathways (p and s, lower) enrichment of predicted target genes expressed in tumor cells. (t) Scatter plot shows Spearman correlation between percentage of cluster1 malignant cells (n = 1546) and *POSTN* expression level in *POSTN*$^{+}$ fibroblasts (n = 3990). Data represent mean ± SD. *P* values were calculated by Fisher's test and were adjusted by FDR in g, k, p, and s, by two-sided Wilcoxon signed-rank test in l, and by two-sided log-rank test in d. Source data are provided as a Source Data Fig. S4a-t.

Supplementary Fig5

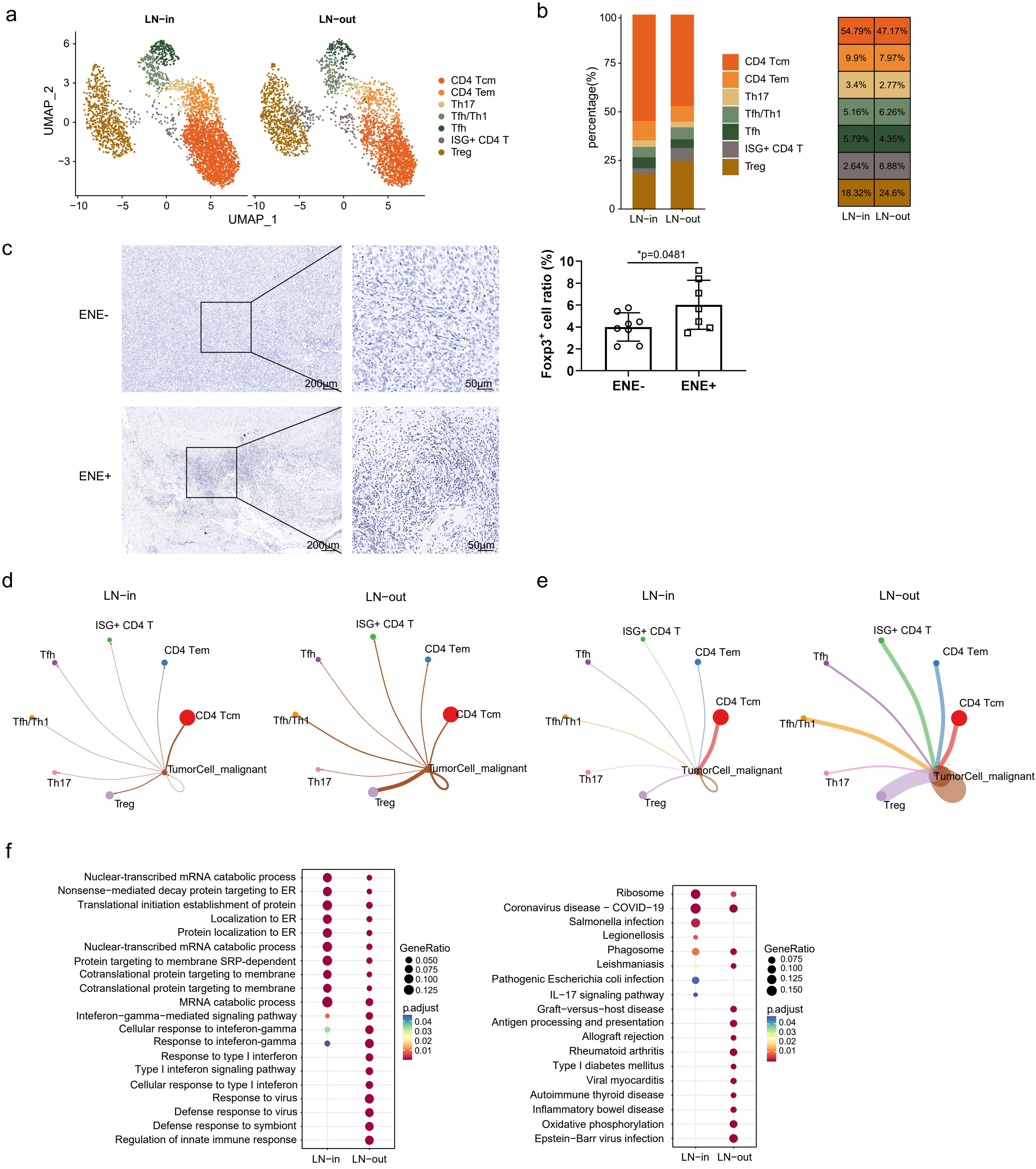

(To be continued)

Supplementary Fig5

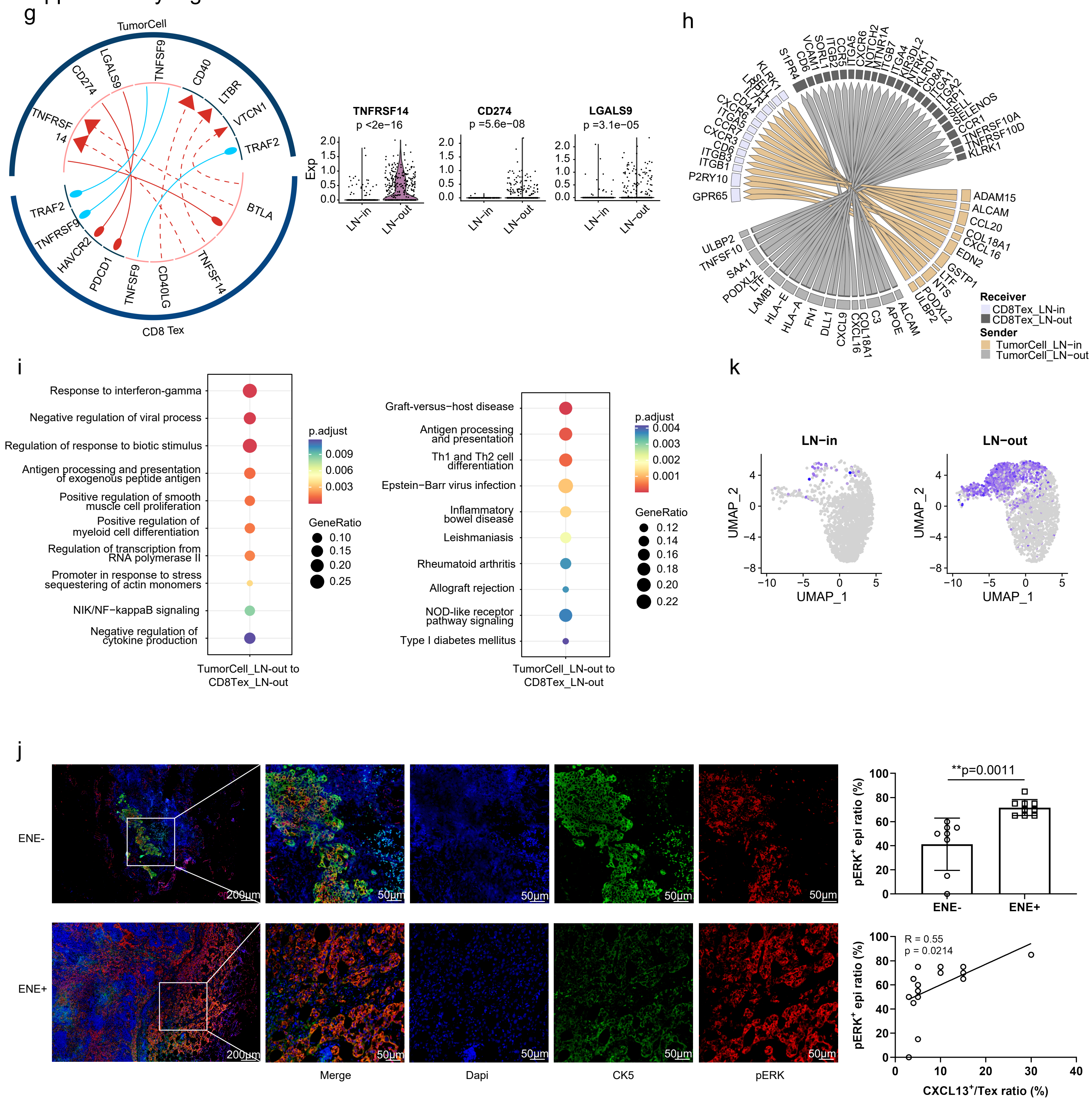

Supplementary Fig5. Interaction between T cells and tumor cells during extranodal extension, related to Figure 5. (a-b) UMAP plot (a) and bar chart (b) show the proportion differences of $CD4^+$ T cell subclusters between LN-out and LN-in samples (3820 cells from 3 LN-in samples and 3036 cells from 2 LN-out samples). (c) Representative IHC staining of Foxp3 in lymph nodes. Scale bar, 200 μm and 50 μm. Proportion of $Foxp3^+$ cell is compared between ENE^- and ENE^+ samples and is shown in the right (n=8 and 7 for each group). (d-e) Circle plots show cell-cell interaction strength differences of tumor cells to $CD4^+$ T cell subclusters (d) and $CD4^+$ T cell subclusters to tumor cells (e) in LN-in and LN-out samples. (f) Bubble plots show representative GO (left) and KEGG pathways (right) enrichment of CD8 Tex cell DEGs between LN-out and LN-in samples. (g) Circle plot and violin plots show representative ligand-receptor pairs between CD8 Tex cells and tumor cells in LN-out samples using iTALK algorithm. Malignant epithelial: 263 cells from LN-in and 360 cells from LN-out samples. (h) Circle plot shows representative ligand-receptor pairs between CD8 Tex cells and tumor cells in LN-out samples. (i) Representative GO (left) and KEGG pathways (right) enrichment of the predicted target genes in CD8 Tex cells. (j) Representative mIHC staining of lymph node samples**.** Dapi (blue), CK5 (green), and pERK (red). Scale bar = 50 μm. Proportion of $pERK^+$ Epi in LN-in and LN-out samples is shown in the right, upper panel (n = 8 and 9). Pearson correlation of *CXCL13*$^+$ Tex ratio with $pERK^+$ epi ratio is shown in the right, lower panel (n = 17). (k) Feature plots show *CXCL13* expression level in $CD8^+$ T cells in LN-in and LN-out samples (2079 cells from 3 LN-in samples and 2878 cells from 2 LN-out samples). Data represent mean ± SD. *P* values were calculated by two-side Student's *t*-test in c and j, by Fisher's test and were adjusted by FDR in f and i, and by two-sided Wilcoxon signed-rank test in g. Source data are provided as a Source Data Fig. S5a-k.

Supplementary Fig6

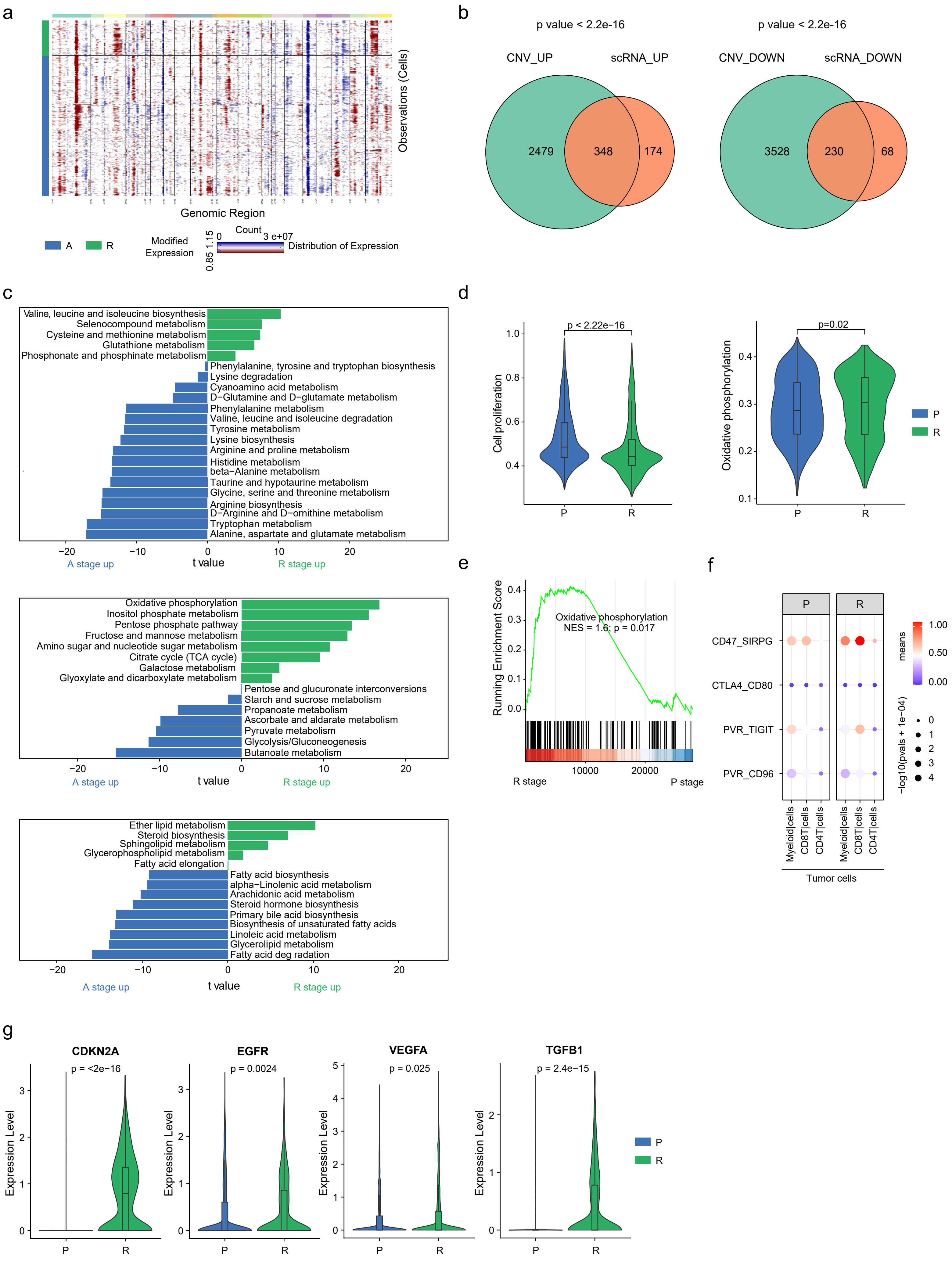

Supplementary Fig6. Characteristics of malignant epithelial cells in recurrent tumors, related to Figure 6. (a) Inferred large-scale CNVs between cells in A and R stages. Rows correspond to individual cells between stage R and A samples (color bars on the left) and columns correspond to genes ordered by chromosomal location. (b) Venn plots show the 348 overlapping genes between inferred copy number gain genes and up-regulated genes from scRNA DEGs (the left panel), the 230 overlapping genes between inferred copy number loss genes and down-regulated genes from scRNA DEGs (the right panel). (c) Bar chart shows the enrichment of specific metabolic pathways (upper panel: carbonate metabolism, middle panel: lipid metabolism, lower panel: amino acid metabolism), based on the KEGG gene set of upregulated genes, in A or R stage malignant cells. (d) Violin plots show the cell proliferation scores from Sun *et.al* and oxidative phosphorylation scores of malignant cells in P (n = 1650) and R (n = 789) stage. (e) GSEA of oxidative phosphorylation pathway between P and R stages. (f) Bubble plots show the interaction between malignant cells and myeloid cells, $CD4^+$, and $CD8^+$ T cells, based on selected ligand and receptor pairs. These scores are normalized expression level, and the sizes of the bubbles indicate the significance of the interactions, calculated by CellPhoneDB. (g) Violin plots show the expression of selected genes in malignant cells in P and R stage. Data from GSE234933 [9] is analyzed for (d, f, and g) and data from GSE173855 [10] is analyzed for (e). Data represent mean ± SD. *P* values were calculated by Fisher's test in b, by two-sided Wilcoxon signed-rank test in d-g, and was calculated based on the interaction and the normalized cell matrix achieved by Seurat Normalization in f. Source data are provided as a Source Data Fig. S6b-g.

Supplementary References

1. Puram SV, Tirosh I, Parikh AS, Patel AP, Yizhak K, Gillespie S, et al. Single-Cell Transcriptomic Analysis of Primary and Metastatic Tumor Ecosystems in Head and Neck Cancer. Cell. 2017;171(7).
2. Cao S, Wendl MC, Wyczalkowski MA, Wylie K, Ye K, Jayasinghe R, et al. Divergent viral presentation among human tumors and adjacent normal tissues. Sci Rep. 2016;6:28294.
3. Elbasir A, Ye Y, Schäffer DE, Hao X, Wickramasinghe J, Tsingas K, et al. A deep learning approach reveals unexplored landscape of viral expression in cancer. Nat Commun. 2023;14(1):785.
4. Elyada E, Bolisetty M, Laise P, Flynn WF, Courtois ET, Burkhart RA, et al. Cross-Species Single-Cell Analysis of Pancreatic Ductal Adenocarcinoma Reveals Antigen-Presenting Cancer-Associated Fibroblasts. Cancer Discovery. 2019;9(8):1102-23.
5. Galbo PM, Zang X, Zheng D. Molecular Features of Cancer-associated Fibroblast Subtypes and their Implication on Cancer Pathogenesis, Prognosis, and Immunotherapy Resistance. Clinical Cancer Research : an Official Journal of the American Association For Cancer Research. 2021;27(9):2636-47.
6. Cheng S, Li Z, Gao R, Xing B, Gao Y, Yang Y, et al. A pan-cancer single-cell transcriptional atlas of tumor infiltrating myeloid cells. Cell. 2021;184(3).
7. Quah HS, Cao EY, Suteja L, Li CH, Leong HS, Chong FT, et al. Single cell analysis in head and neck cancer reveals potential immune evasion mechanisms during early metastasis. Nat Commun. 2023;14(1):1680.
8. Puram SV, Mints M, Pal A, Qi Z, Reeb A, Gelev K, et al. Cellular states are coupled to genomic and viral heterogeneity in HPV-related oropharyngeal carcinoma. Nature Genetics. 2023;55(4):640-50.
9. Bill R, Wirapati P, Messemaker M, Roh W, Zitti B, Duval F, et al. CXCL9:SPP1 macrophage polarity identifies a network of cellular programs that control human cancers. Science. 2023;381(6657):515-24.
10. Weber P, Künstner A, Hess J, Unger K, Marschner S, Idel C, et al. Therapy-Related Transcriptional Subtypes in Matched Primary and Recurrent Head and Neck Cancer. Clinical Cancer Research : an Official Journal of the American Association For Cancer Research. 2022;28(5):1038-52.
