# Supplementary material for: Single cell deciphering of progression trajectories of the tumor ecosystem in head and neck cancer
Source: Nat Commun. 2024 Mar 22;15:2595. doi: 10.1038/s41467-024-46912-6 (PMC10959966; doi:10.1038/s41467-024-46912-6)
Supplement: Supplementary file 3 — Description of Additional Supplementary Files [file 41467_2024_46912_MOESM3_ESM.pdf]

Supplementary Datasets

Supplementary Data 1. Clinical information of scRNA-seq cohort..

Supplementary Data 2. Clinical information of validation cohort 1.

Supplementary Data 3. Clinical information of validation cohort 2.

Supplementary Data 4. Signature genes from other studies.
