# Supplementary material for: Single cell deciphering of progression trajectories of the tumor ecosystem in head and neck cancer
Source: Nat Commun. 2024 Mar 22;15:2595. doi: 10.1038/s41467-024-46912-6 (PMC10959966; doi:10.1038/s41467-024-46912-6)
Supplement: Supplementary file 5 — Reporting Summary [file 41467_2024_46912_MOESM5_ESM.pdf]

Corresponding author(s): He Y
Last updated by author(s): Mar 6, 2024

Reporting Summary

Nature Portfolio wishes to improve the reproducibility of the work that we publish. This form provides structure for consistency and transparency in reporting. For further information on Nature Portfolio policies, see our Editorial Policies and the Editorial Policy Checklist.

Statistics

For all statistical analyses, confirm that the following items are present in the figure legend, table legend, main text, or Methods section.

n/a	Confirmed	
☐	☒	The exact sample size (*n*) for each experimental group/condition, given as a discrete number and unit of measurement
☐	☒	A statement on whether measurements were taken from distinct samples or whether the same sample was measured repeatedly
☐	☒	The statistical test(s) used AND whether they are one- or two-sided<br>*Only common tests should be described solely by name; describe more complex techniques in the Methods section.*
☐	☒	A description of all covariates tested
☐	☒	A description of any assumptions or corrections, such as tests of normality and adjustment for multiple comparisons
☐	☒	A full description of the statistical parameters including central tendency (e.g. means) or other basic estimates (e.g. regression coefficient) AND variation (e.g. standard deviation) or associated estimates of uncertainty (e.g. confidence intervals)
☐	☒	For null hypothesis testing, the test statistic (e.g. *F*, *t*, *r*) with confidence intervals, effect sizes, degrees of freedom and *P* value noted<br>*Give P values as exact values whenever suitable.*
☒	☐	For Bayesian analysis, information on the choice of priors and Markov chain Monte Carlo settings
☒	☐	For hierarchical and complex designs, identification of the appropriate level for tests and full reporting of outcomes
☐	☒	Estimates of effect sizes (e.g. Cohen's *d*, Pearson's *r*), indicating how they were calculated

Our web collection on statistics for biologists contains articles on many of the points above.

Software and code

Policy information about availability of computer code

Data collection	No special or proprietary software was used.
Data analysis	Softwares were used as follows:<br>Seurat v4.1.1<br>Harmony v0.1.0<br>ClusterProfiler v4.0.5<br>magrittr v2.0.3<br>dplyr v1.0.8<br>ggplot2 v3.3.5<br>stringr v1.4.0<br>RColorBrewer v1.1.3<br>readr v2.0.0<br>infercnv v1.8.1<br>Monocle v2.20.0<br>velocyto.R v1.0.8<br>SCENIC v1.1.3<br>CellChat v1.5.0<br>NicheNet v1.1.0<br>survival v3.3.1<br>GSVA v1.40.1

maxstat v0.7.25
3DHISTECH

For manuscripts utilizing custom algorithms or software that are central to the research but not yet described in published literature, software must be made available to editors and reviewers. We strongly encourage code deposition in a community repository (e.g. GitHub). See the Nature Portfolio guidelines for submitting code & software for further information.

Data

Policy information about availability of data

All manuscripts must include a data availability statement. This statement should provide the following information, where applicable:

- Accession codes, unique identifiers, or web links for publicly available datasets
- A description of any restrictions on data availability
- For clinical datasets or third party data, please ensure that the statement adheres to our policy

The bulk RNA-seq publicly available data used in this study are available in the TCGA portal (http://gdac.broadinstitute.org/) and the Gene Expression Omnibus under accession code GSE173855 (https://www.ncbi.nlm.nih.gov/geo/query/acc.cgi?acc=GSE173855). The processed publicly available scRNA-seq data used in this study are available in the Gene Expression Omnibus under accession code GSE188737 (https://www.ncbi.nlm.nih.gov/geo/query/acc.cgi?acc=GSE188737), GSE182227 (https://www.ncbi.nlm.nih.gov/geo/query/acc.cgi?acc=GSE182227), and GSE234933 (https://www.ncbi.nlm.nih.gov/geo/query/acc.cgi?acc=GSE234933).
The raw data of single-cell RNA-seq generated in this study were deposited in Genome Sequence Archive (GSA) with accession ID HRA004648. Since these data are related to human genetic resources, raw data can be obtained directly by requesting and following the GSA guidelines for academic use at https://ngdc.cncb.ac.cn/gsa-human/browse/HRA004648 after the user log in to the GSA database with the email address of the academic institution. The request will be responded to within two weeks. Once access is granted, users have six months to download the data. The guidance for making a data access request of GSA for humans can be downloaded from https://ngdc.cncb.ac.cn/gsa-human/document/GSA-Human_Request_Guide_for_Users_us.pdf.
The signature gene lists from other studies are listed in Supplementary Data 4. The remaining data are available within the Article, Supplementary Information or Source Data file.

Research involving human participants, their data, or biological material

Policy information about studies with human participants or human data. See also policy information about sex, gender (identity/presentation), and sexual orientation and race, ethnicity and racism.

Reporting on sex and gender	1. 6 out of 13 HNSCC patients are female for scRNA-seq in this study, no sex or gender analysis was carried out.<br>2. 17 out of 53 HNSCC patients are female in validation cohort 1, no sex or gender analysis was carried out.<br>3. 5 out of 20 HNSCC patients are female in validation cohort 2, no sex or gender analysis was carried out.<br>Gender analysis was not performed because we focused on the differences between cancer stages instead of genders.
Reporting on race, ethnicity, or other socially relevant groupings	The research was not involved in race, ethnicity or other socially relevant groupings. The relevant information was not taken under consideration.
Population characteristics	1. The scRNA-seq cohort of 13 HNSCC patients were patients with primary or recurrent HNSCC and samples were collected between July 2020 and May 2021. They were treatment-naive (primary) or treated with surgery (recurrent). Their ages range from 34 to 76, with 7 male patients and 6 female patients. The clinical information is summarized in Supplementary Data1.<br>2. The validation cohort1 of 53 HNSCC patients includes 23 patients with early stage and 30 patients with advanced stage HNSCC. They were treatment-naive. Their ages range from 22 to 86, with 36 male patients and 17 female patients. The clinical information is summarized in Supplementary Data2.<br>3. The validation cohort2 of 20 HNSCC patients includes 10 patients with ENE- lymph node metastasis and 10 patients with ENE+ lymph node metastasis. They were treatment-naive. Their ages range from 22 to 76, with 15 male patients and 5 female patients. The clinical information is summarized in Supplementary Data3.
Recruitment	Our study does not involve patient recruitment. Patients with HNSCC and clinicopathological information were retrospectively retrieved.
Ethics oversight	The study was conducted in accordance with ethical guidelines of U.S. Common Rule. All tissue samples were collected in compliance with the informed consent policy. The study was supervised by Ethics Committee of Shanghai Ninth People's Hospital Affiliated to Shanghai Jiao Tong University School of Medicine.

Note that full information on the approval of the study protocol must also be provided in the manuscript.

Field-specific reporting

Please select the one below that is the best fit for your research. If you are not sure, read the appropriate sections before making your selection.

☒ Life sciences ☐ Behavioural & social sciences ☐ Ecological, evolutionary & environmental sciences

For a reference copy of the document with all sections, see nature.com/documents/nr-reporting-summary-flat.pdf

Life sciences study design

All studies must disclose on these points even when the disclosure is negative.

Sample size	scRNA-seq cohort: n=26, including different HNSCC stages; validation cohort1: n=68, including different HNSCC stages; validation cohort2: n=20, including ENE- and ENE+ sample.<br>No sample size calculation was performed. We followed the routine biological replicate requirement in experiment section, n >= 3 for each group.
Data exclusions	For sequencing data, we excluded low-quality cells if abnormalities exist in (1) cell library sizes; (2) the numbers of expressed genes; (3) the proportion of mitochondrial gene counts. The details of cut-off line could be checked in Methods.
Replication	All replications were successful, and the detailed information was provided in corresponding figure legends.
Randomization	ScRNA-seq cohort, validation cohort 1 and 2 are retrospective cohorts , so randomization is not relevant to our study.
Blinding	Not applicable since there was no specific grouping.

Reporting for specific materials, systems and methods

We require information from authors about some types of materials, experimental systems and methods used in many studies. Here, indicate whether each material, system or method listed is relevant to your study. If you are not sure if a list item applies to your research, read the appropriate section before selecting a response.

Materials & experimental systems

n/a	Involved in the study	
☐	☒	Antibodies
☐	☒	Eukaryotic cell lines
☒	☐	Palaeontology and archaeology
☒	☐	Animals and other organisms
☒	☐	Clinical data
☒	☐	Dual use research of concern
☒	☐	Plants

Methods

n/a	Involved in the study	
☒	☐	ChIP-seq
☒	☐	Flow cytometry
☒	☐	MRI-based neuroimaging

Antibodies

Antibodies used	The following antibodies were used: anti-TFDP1 primary antibody (Proteintech, 11043-1-AP, 1:200), anti-CK5 primary antibody (Abcam, ab52635, 1:200), anti-POSTN primary antibody (Abcam, ab152099, 1:1000), anti-α-SMA antibody (Abcam, ab124964, 1:1000), anti-SPP1 antibody (Abcam, ab214050, 1:1000), anti-CD68 antibody (Abcam, ab955, 1:3000), anti-CXCL13 (Abcam, ab246518, 1:1000), anti-CD8 (CST, #70306, 1:400), anti-PD1 (Abcam, ab216352, 1:50), and anti-pERK (Abcam, ab201015, 1:500)
Validation	All the antibodies used in this study were commercial antibodies and were only used for applications, with validation procedures described on the following sites of the manufacturers.<br>anti-TFDP1 primary antibody (Proteintech, 11043-1-AP, 1:200):Application: IHC; Reactivity: Human,Mouse, Rat<br>anti-CK5 primary antibody (Abcam, ab52635, 1:200):Application: Flow Cyt (Intra), ICC/IF, IHC-P, mIHC, WB; Reactivity: Mouse, Human<br>anti-POSTN primary antibody (Abcam, ab152099, 1:1000):Application: ICC/IF, IHC-P, WB; Reactivity: Mouse, Human<br>anti-α-SMA antibody (Abcam, ab124964, 1:1000): Application: Flow Cyt (Intra), ICC/IF, IHC-P, WB;Reactivity: Mouse, Rat, Human<br>anti-SPP1 antibody (Abcam, ab214050, 1:1000):Application: ICC/IF, IHC-P, IP, WB; Reactivity:Human<br>anti-CD68 antibody (Abcam, ab955, 1:3000):Application: ICC/IF, IHC-P, Mass Cytometry, WB;Reactivity:Human<br>anti-CXCL13 (Abcam, ab246518, 1:1000):Application: IHC-P; Reactivity: Human<br>anti-CD8 (CST, #70306, 1:400):Application: IHC-P; Reactivity: Human<br>anti-PD1 (Abcam, ab216352, 1:50):Application: Flow Cyt (Intra), IHC-P, IP, WB; Reactivity: Human<br>anti-pERK (Abcam, ab201015, 1:500):Application: Dot, ICC/IF, IHC-P, IP, WB; Reactivity: Mouse, Rat, Human

Eukaryotic cell lines

Policy information about cell lines and Sex and Gender in Research

Cell line source(s)	Cal27 and SCC9 cells were obtained from American Type Culture Collection.
Authentication	The cells were authenticated based on the morphology under microscope and growth rate.
Mycoplasma contamination	Cell lines were tested negative for mycoplasma contamination.

Commonly misidentified lines (See ICLAC register)	No misidentified lines were used.

Plants

Seed stocks	*Report on the source of all seed stocks or other plant material used. If applicable, state the seed stock centre and catalogue number. If plant specimens were collected from the field, describe the collection location, date and sampling procedures.*
Novel plant genotypes	*Describe the methods by which all novel plant genotypes were produced. This includes those generated by transgenic approaches, gene editing, chemical/radiation-based mutagenesis and hybridization. For transgenic lines, describe the transformation method, the number of independent lines analyzed and the generation upon which experiments were performed. For gene-edited lines, describe the editor used, the endogenous sequence targeted for editing, the targeting guide RNA sequence (if applicable) and how the editor was applied.*
Authentication	*Describe any authentication procedures for each seed stock used or novel genotype generated. Describe any experiments used to assess the effect of a mutation and, where applicable, how potential secondary effects (e.g. second site T-DNA insertions, mosiacism, off-target gene editing) were examined.*
